# Dual HER2 Blockade: An Emerging Option in Metastatic Biliary Tract Cancer?

**DOI:** 10.3390/medicina57121301

**Published:** 2021-11-27

**Authors:** Angela Dalia Ricci, Alessandro Rizzo

**Affiliations:** Department of Experimental, Diagnostic and Specialty Medicine, S. Orsola-Malpighi University Hospital, 40138 Bologna, Italy; rizzo.alessandro179@gmail.com

Biliary tract cancer (BTC) includes a heterogeneous group of aggressive and rare hepatobiliary malignancies, including gallbladder cancer, ampullary carcinomas, intrahepatic cholangiocarcinoma (iCCA), and extrahepatic cholangiocarcinoma, further subclassified into distal (dCCA) and perihilar cholangiocarcinoma (pCCA) [1,2]. These anatomical subgroups arise from distinct locations of the biliary tree and present important differences in etiology, biology, epidemiology, and prognosis [3,4]. We have recently witnessed a revolution leading to the advent of next-generation sequencing, something that has paved the way towards the identification of the molecular landscape of BTC [5,6]. Thus, several potentially druggable alterations have been identified and a number of molecularly targeted agents are under assessment in this setting, since approximately 50% of BTC patients are deemed to present potentially actionable aberrations [7,8]. In fact, several potential therapeutic targets have been described and investigated, including fibroblast growth factor receptor (FGFR) fusions, mutations in isocitrate dehydrogenase (IDH)-1, and BRAF mutations [9,10,11]. However, results regarding HER2 inhibitors have been controversial so far [12,13].

In *The Lancet Oncology*, Javle and colleagues have recently reported the results of the BTC cohort of the MyPathway HER2 non-randomized phase 2a multiple basket trial, demonstrating preliminary signs of activity of dual HER2-targeted combination of pertuzumab–trastuzumab in previously treated metastatic BTC with HER2 amplification and/or overexpression [14]. Despite the small sample size and the single-arm design of the study, the promising dual anti-HER2 blockade demonstrated an objective response rate of 23% (95% CI 11–39), a disease control rate of 51% (95% CI 35–68), and a 1-year overall survival (OS) rate of 50% in this patient population, with a manageable safety profile. Notably, this benefit appears particularly pronounced in gallbladder and ampullary carcinomas, where median OS reached 14.2 and 17.1 months, respectively, although caution should be exercised due to the small proportion of enrolled patients. In addition, exploratory analyses investigated the emerging role of circulating tumor DNA (ctDNA) and its pioneering implication in monitoring treatment response [15]. This study addresses an important research question, since there is a lack of data to guide treatment decisions beyond first-line treatment in these rare malignancies. Indeed, recent findings from ABC-06 showed that systemic therapy with second-line modified oxaliplatin and 5-fluorouracil (mFOLFOX) plus active symptom control (ASC) confers a modest survival benefit in comparison with ASC alone [16].

Nevertheless, some elements deserve discussion. Traditionally, immunohistochemistry (IHC) and fluorescence in situ hybridization (FISH) are the most commonly used techniques for assessing HER2 status across solid tumors. As concerns BTC, there are no standardized methods to determine HER2 positivity so far and the majority of previous reports have been conducted using IHC and following gastric cancer scoring criteria [17]. In MyPathway, BTC patients with HER2 amplification and or overexpression were selected on the basis of a broad spectrum of non-mutually exclusive methods: IHC, FISH, chromogenic in situ hybridization, and next-generation sequencing. However, given the disappointing results reported in this analysis about the efficacy of anti-HER2 dual blockade in patients with HER2 amplification without overexpression, with all four patients whose disease progressed within 2 months, the question also arises whether IHC testing could be replaced by innovative sequencing technologies both in future trials and, more importantly, in clinical practice.

In conclusion, the MyPathway trial provides a relevant contribution to the field and represents a new hope for a relevant percentage of BTC patients harboring HER2 overexpression and/or amplification, but future research is needed to confirm these novel findings and to better understand the role of dual HER2 blockade in this setting.

## Data Availability

Not applicable.
